# Optimization and Validation of a Chromatographic Method for the Quantification of Isoniazid in Urine of Tuberculosis Patients According to the European Medicines Agency Guideline

**DOI:** 10.3390/antibiotics7040107

**Published:** 2018-12-11

**Authors:** Pooja Mishra, Jaume Albiol-Chiva, Devasish Bose, Abhilasha Durgbanshi, Juan Peris-Vicente, Samuel Carda-Broch, Josep Esteve-Romero

**Affiliations:** 1 Department of Chemistry, University Doctor Harisingh Gour Vishwavidyalaya (A Central University), Sagar, Madhya Pradesh 470003, India; mishrapooja2013@gmail.com; 2 Química Bioanalítica, Departament de Química Física i Analítica, Escola Superior de Tecnologia i Ciències Experimentals, Universitat Jaume I, 12071 Castelló, Spain; albiolj@uji.es (J.A.-C.); juan.peris@uji.es (J.P.-V.); scarda@uji.es (S.C.-B.); josep.esteve@uji.es (J.E.-R.); 3 Department of Criminology and Forensic Science, Harisingh Gour Vishwavidyalaya (A Central University), Sagar, Madhya Pradesh 470003, India; devonebose@gmail.com

**Keywords:** direct injection, drug, micellar, tuberculosis, urine, validation

## Abstract

Isoniazid is a drug that is widely used against tuberculosis. However, it shows high interpatient variability in metabolism kinetics and clinical effect, which complicates the prescription of the medication and jeopardizes the success of the therapy. Therefore, in a specific patient, the pharmacokinetics of the drug must be elucidated to decide the proper dosage and intake frequency to make the drug suitable for therapeutic drug monitoring. This can be performed by the quantification of the drug in urine as this process is non-invasive and allows the effects of long-time exposure to be inferred. The paper describes the development of a micellar liquid chromatographic method to quantify isoniazid in urine samples. Extraction steps were avoided, making the procedure easy to handle and reducing the waste of toxic organic solvents. Isoniazid was eluted in less than 5 min without interference from other compounds of the urine using a mobile phase containing 0.15 SDS–12.5% 1-propanol (*v*/*v*)–Na_2_HPO_4_ 0.01 M buffered at pH 7, running at 1 mL/min under isocratic mode through a C18 column with the detection wavelength at 265 nm. The method was validated by following the requirements of the Guidelines on Bioanalytical Method Validation issued by the European Medicines Agency (EMA) in terms of selectivity, calibration curve (*r*^2^ = 0.9998 in the calibration range (0.03–10.0 μg/mL), limit of detection and quantification (10 and 30 ng/mL respectively), precision (<16.0%), accuracy (−0.9 to +8.5%), carry-over, matrix effect, and robustness. The developed method was applied to quantify isoniazid in urine samples of patients of an Indian hospital with good results. The method was found to be useful for routine analysis to check the amount of isoniazid in these patients and could be used in its therapeutic monitoring.

## 1. Introduction

**Figure 1:**
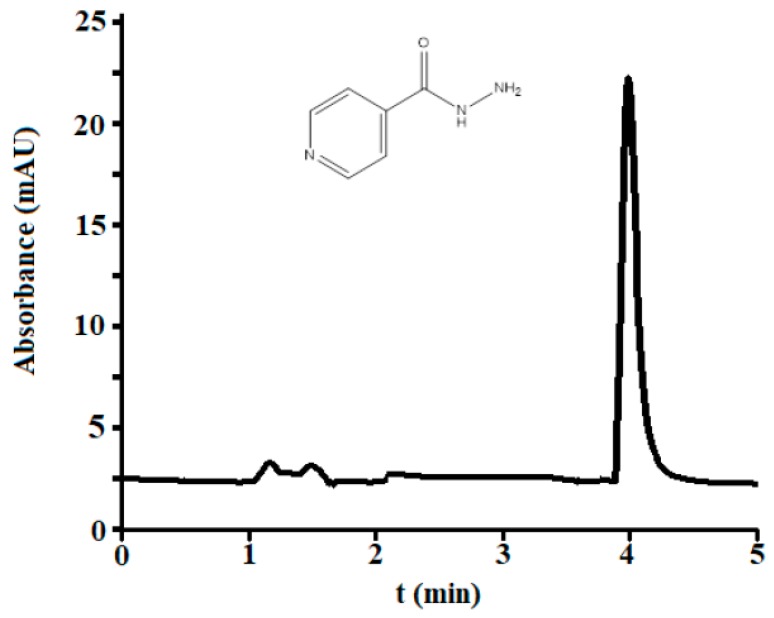
Chromatogram of standard isoniazid (1 μg/mL) obtained under the optimal conditions. Structure of isoniazide (MW = 137 g/mol).

Isoniazid (Figure 1) is known for its antibacterial activity, which is caused by the inhibition of the biosynthesis of mycolic acid. It effectively kills bacteria that are actively growing in the body tissues with fluid content just within the first two weeks of treatment [1]. Once administrated, isoniazid is well absorbed and distributed rapidly throughout the fluid part of the body. It is one of the drugs that is normally used in the treatment of tuberculosis and involves two phases. The initial stage lasts 2 months, and the main objective is to kill as many bacteria as possible. Due to the above-mentioned reason, in the first two-month period, rifampicin, pyrazinamide, isoniazid, and ethambutol are given to the patient. After the completion of the first stage, rifampicin and isoniazid are administered until the end of second stage of treatment which lasts another four months. Once ingested by the patient, isoniazid is primarily metabolized in the liver to acetyl isoniazid by acetyl transferase [2,3]. The acetylation is controlled genetically, and it varies from patient to patient. Some of the patients are slow acetylators and some others are fast acetylators. Many researchers have reported that in fast acetylators, 90% of the drug is excreted as acetylisoniazid, resulting in short lasting effects of the drug which may lead to therapeutic failure [4]. In slow acetylators, 67% of the drug is excreted in acetylated form resulting in a higher blood concentration of the parent drug, thus leaving patients prone to more severe side effects, such as peripheral neuropathy, which is the most common Central Nervous System (CNS)-related toxic effect [5]. Due to its complex metabolic process, there is always a need for its monitorization in urine, especially to decide the proper dosage which normally varies from person to person depending on their metabolism, as it depends on highly-variable factors, like genetics, habits, health, physiological state, and the environment, among others.

Several methods for the detection and quantification of isoniazid from biological fluids and pharmaceuticals have been developed. These approaches use thin layer chromatography (TLC), high performance liquid chromatography) (HPLC), or liquid and gas chromatography coupled to mass spectrometry detectors (GC/MS, LC/MS) [6,7,8,9,10,11,12,13]. However, these techniques require sophisticated and delicate instrumentation and trained laboratory staff and are relatively expensive in terms of maintenance and purchase. Apart from this, these methods are time-consuming, and require cumbersome pretreatment and a large amount of hazardous chemicals. Therefore, they are not suitable for routine clinical use [14,15].

Micellar liquid chromatography (MLC), using a C18 column and the anionic surfactant sodium dodecyl sulfate, is a reverse-phase liquid chromatographic subtechnique that has great potential to overcome the above-mentioned problems [14]. The major advantage of this technique is that it does not require any pretreatment for biological samples, as proteins and other endogenous compounds are denatured and solubilized by the micellar environment and can be harmlessly injected into the column. Besides, they are usually eluted at the front of the chromatogram, thus reducing the risk of overlapping with the analytes, even the barely retained ones [15]. Many drugs, such as antitumorals, antiepileptics, antimalarials, antibiotics, and psychoactive compounds, in biological fluids, such as plasma, serum, and urine have already been successfully analyzed by MLC [16,17,18,19,20]. The experimental protocol is expedited to a simple dilution, usually 1/5 *v*/*v*, filtration, and direct injection in the column. The drugs are resolved from the matrix and endogenous compounds using a hybrid micellar mobile phase running under isocratic mode within a reasonable time period. 

The aim of the work is to develop a simple, reliable, cost-effective, and rapid method for the determination of isoniazid in urine that can easily be used for the regular monitorization of isoniazid in tuberculosis patients in hospitals. It is validated by the Guidelines of the European Medicines Agency in order to verify its analytical performances, and applied to incurred samples [21,22].

## 2. Experimental

### 2.1. Chemical and Reagents

Isoniazid (purity >99.0%) was purchased from Merck (Darmstadt, Germany). Sodium dodecyl sulphate (SDS, 99% purity), sodium dihydrogen phosphate, and sodium acetate (analytical grade) were purchased from Himedia Laboratories Private Limited (Mumbai, India). Hydrochloric acid, sodium hydroxide, and HPLC grade 1-propanol were provided by Rankem, RFCL Limited (New Delhi, India). All the solutions were filtered through 0.45 µm nylon membrane filters from Micron Separation (Westboro, MA, USA). An ultrasonic bath (Model Ultrasons-H; Selecta, Barcelona, Spain) was used to achieve the solubilization of the solids. Ultrapure water was in-lab elaborated from deionized water using an Ultrapure water generator device, Simplicity UV (Millipore S.A.S., Molsheim, France). This water was used to prepare the aqueous solutions.

### 2.2. Apparatus and Instrumentation

The pH of the mobile phase was measured by using a digital pH meter pH-102/103 Contech, Instruments Limited, (Mumbai, India). The analytical weighing machine used was from Mettler Toledo India Private Limited (Mumbai, India).

Chromatographic analyses were performed on Shimadzu Prominence HPLC System, Shimadzu Corporation, (Kyoto, Japan) equipped with an isocratic pump LC-20 AT, an autosampler SIL-20AC, and a diode array detector SPD-M20 A (190–800 nm). The column used for the analysis was a SPHER-100 C_18_ 100A (250 mm × 4.6 mm × 5 µm particle size) from Princeton Chromatography INC (Cranbury, NJ, USA). The mobile phase was an aqueous solution of 0.15 M SDS–12.5% 1-propanol–0.01 M Na_2_HPO_4_ 0.01 M buffered at pH 7, running at 1 mL/min under isocratic mode at room temperature. The injection volume and the absorbance detection wavelength were 20 μL and 265 nm, respectively. All injected solutions, either standard of diluted samples, had been previously filtered through 0.45-μm nylon membrane filters (Micron Separations, Westboro, MA, USA) by pushing with a 3-mL syringe. The processed samples were thrown away after injection. The special care required for the HPLC system when working with micellar mobile phases is detailed in [23].

A personal computer in which Shimadzu LC Solution software version 1.22 SP1 was installed was used to control the instrumentation, register the signal, and determine the main chromatographic parameters: retention time (t_R_), retention factor (*k*), efficiency (number of theoretical plates, N), and the asymmetry (B/A). These parameters were calculated as indicated in [24]. 

### 2.3. Preparation of Solutions

Mobile phases were prepared by accurately weighing the proper amount of SDS and sodium dihydrogen phosphate salt, which were then dissolved in HPLC grade water with the aid of a magnetic stirrer. The pH was adjusted by adding drops of NaOH (0.1 M) or HCl (0.1 M). Thereafter, the organic solvent was introduced to reach the desired proportion, and then the volumetric flask was filled up with water, ultrasonicated for 5 min, and filtered through a through 0.45-μm nylon membrane filters (Micron Separations, Westboro, MA, USA) with the help of a vacuum pump.

Standard stock solution of isoniazid 100 µg/mL^−1^ was prepared by dissolving it in a small amount of water, following by ultrasonication (5 min). Working solutions were made by successive dilutions in the mobile phase.

### 2.4. Sample Collection and Processing

Urine samples from tuberculosis patients and healthy volunteers (taking no medications) were collected in glass tubes and stored at −20 °C, after consent regulated by the Ethical Committee of the Sagar’s Hospital (ethics approval code 2018-ICMR-001027, approved by the Indian Council for Medical Research). The investigations were carried out following the ethical rules of the Declaration of Helsinki 1975, revised in 2013. For confidentiality reasons, no personal or clinical information about the patients or the healthy volunteers (except that indicated below) was provided from the Hospital. The laboratory will not transmit any information to other institutions beyond that included in the publication and will destroy all of the urine samples and chromatogram files one year after publication of the paper.

Urine from healthy volunteers was drug-free. A matrix-matched blank sample was constructed by mixing equivalent volumes from six volunteers—three men and three women, including one individual in their twenties, another in their thirties and the last one in their forties for each gender. This sample was used as “blank sample” throughout the entire research.

Before being injected into the HPLC system, the preserved aliquots were brought to room temperature and 1/5 diluted with the optimized mobile phase without further pretreatment [14]. For spiked samples, the appropriate volume of a working standard solution was added before dilution. 

## 3. Results and Discussion

### 3.1. Optimization of Micellar Chromatographic Conditions

The tandem C18 column/sodium dodecyl sulfate was selected, as it has been largely proven as the best option to resolve drugs in biological fluids [14]. The standard mobile phase flow rate and program were used: 1 mL/min and isocratic. The optimized parameters were the pH, the concentration of SDS, the nature and proportion of organic solvent, and the wavelength absorbance detection. The assays were performed by the analysis of a working solution of 1 μg/mL of isoniazid. 

#### 3.1.1. Optimization of the pH

The study was restricted to the working range of the column: 2.5–7.5. Different mobile phases containing 0.15 M SDS–0.01 M buffer salt at pH 3 (phosphate), 5 (acetic), and 7 (phosphate) were assayed. For pH 3, the retention time was too long (15.9 min) and a large tailing appeared and then was discarded. The results were similar for pH 5 and 7; the analyte was eluted at 8.3 min, with an adequate peak shape. The optimal pH was set to 7 as this pH is far enough away from the pKas, has negligible concentrations of the acid/basic forms, and due to being neutral, is less aggressive than acidic conditions for the alkyl C18-silica bonds.

Isoniazid contains three ionizable nitrogen atoms: pyridine N (pKa = 1.8); hydrazine–NH (pKa = 3.6) and hydrazine NH_2_ (pKa = 10.8) [25]. Therefore, isoniazid has a charge of +2 from pH 2.5 to 3.6; and +1 from 3.6 to 7.5. In both cases, the analyte would be attracted to the sulfate anionic groups located at the outer layer of the modified stationary phase and the micellar pseudo phase by electrostatics. However, for a positively bicharged molecule, this attraction would be very strong, slowing down its movement in the bulk mobile phase. This explains the longer retention and peak broadening and distortion. 

Under these conditions, the interactions between the analyte and both the stationary phase and the micellar pseudophase were mainly due to electrostatics, as isoniazid would barely interact with the hydrophobic C18 chains of both environments due to its polarity (log P = −0.7 [26]).

#### 3.1.2. Optimization of SDS and Organic Modifier

In order to accelerate the elution and improve the peak shape, the addition of an organic modifier was considered. The alcohol 1-propanol was selected, as 1-butanol and 1-pentanol are more hydrophobic and would excessively increase the elution strength of the mobile phase. The minimal (2.5%), average (7.5%), and maximal proportions (12.5%) of 1-propanol recommended for MLC were assayed, and the respective experimental values of (t_R_, min; N; B/A) were (5.1; 2817; 3.1), (4.3; 3518; 1.8), and (4.0; 3912; 1.2). In all cases, the retention times were enough to avoid overlapping with the front of the chromatogram.

According to the criteria of maximal efficiency-minimal analysis time, the optimal mobile phase was 0.15 M SDS–12.5% 1-propanol–0.01 M phosphate salt buffered at pH 7 (Figure 1).

#### 3.1.3. Selection of the Optimal Detection Wavelength

Structurally, isoniazid has a pyridine ring, a carboxylic group, and a hydrazine group (Figure 1), thus having a high degree of extended conjugation, resulting in its absorption in the UV region. The absorbance spectrum was measured using an UV Visible Spectrophotometer using the mobile phase as a solvent, and the λ_max_ was 265 nm. The UV absorbance spectrum was also taken using the photodiode array detector (PDA) of the chromatogram during an analysis at different times of the isoniazid peak: the maximal height, the front/tail half-height, and the front/tail at 0.1-height points. All were alike and had a similar shape to that obtained under static conditions. 

### 3.2. Method Validation

The method was validated following the Guidelines on Bioanalytical Method Validation issued by the European Medicines Agency (EMA) [21,22]. The evaluated validation parameters were the selectivity, calibration curve, linearity, lower and upper limits of quantification, precision, accuracy, carry-over, matrix effect [21], and limits of detection and robustness [22]. Unless otherwise specified, the validation was performed by using the matrix matched blank, prepared as described in 2.4.

#### 3.2.1. Selectivity

**Figure 2:**
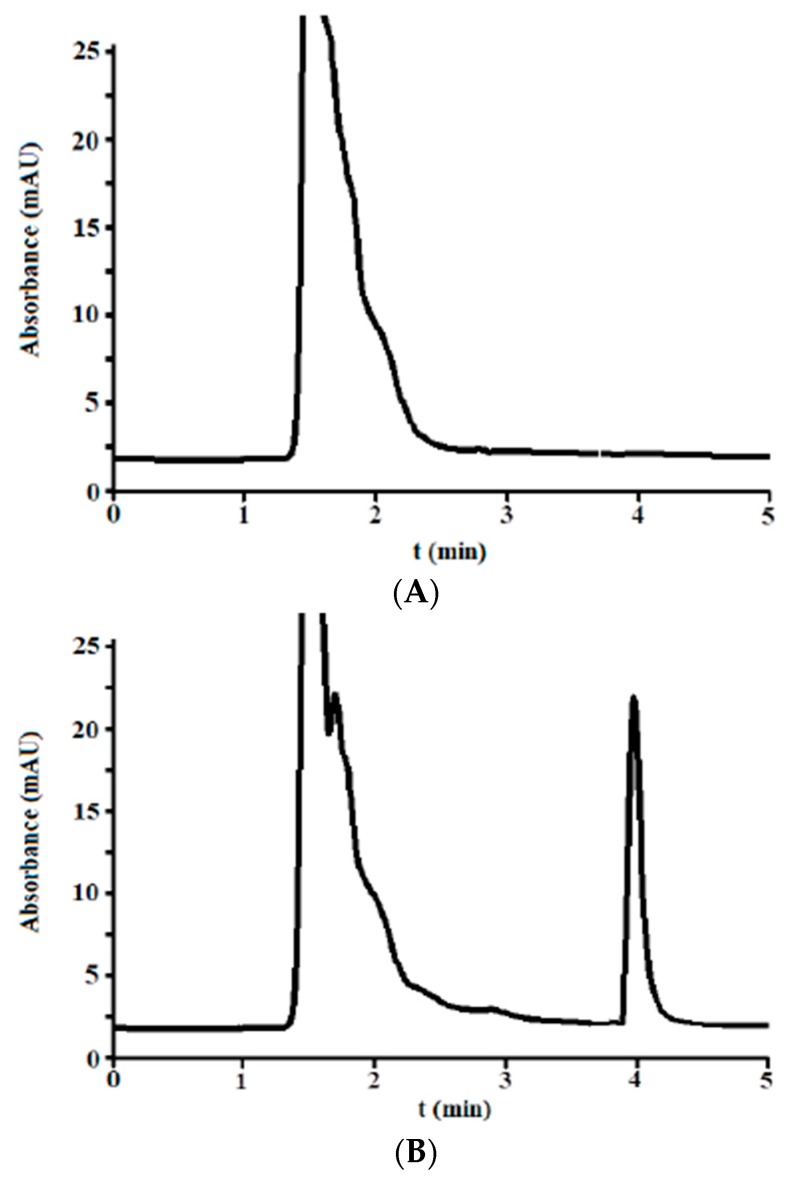
Chromatogram of blank urine from a healthy donor using the optimum chromatographic condition: (**A**) blank and (**B**) fortified at 5 μg/mL (by adding isoniazid to the blank sample).

A selectivity study was performed to verify the presence of other endogenous compound co-eluting out near or with the analyte of interest. Ten blank urine samples were injected using the selected optimum conditions, before and after fortification (addition of isoniazid) at 5 μg/mL. The obtained chromatograms for one case are shown in Figure 2A (blank) and Figure 2B (fortified). 

Several high and broad peaks corresponding to endogenous compounds of urine were eluted from the dead time to nearly 3.0 min in the blanks. This broad band was also observed in the fortified samples. At higher times, the signal was at a fairly stable baseline. No peaks were observed near or at the window time of the analyte. In order to assess the peak purity, the fortified samples were compared to those obtained in Section 3.1.2 by overlaying, and no distortion was noticed. Besides, the absorbance spectra were taken at the same times as described in Section 3.1.3 and showed a similar shape. 

Therefore, the analyte can be reliably identified in the urine matrix, and the peak at 4.0 min exclusively corresponds to isoniazid. 

#### 3.2.2. Linearity and Sensitivity

Calibration curves were obtained for nine different concentrations of isoniazid (six replicates), equally distributed in the range of 0.03 (lower limit of quantification, LLOQ) to 10 μg/mL (upper limit of quantification, ULOQ). The calibration range was selected to cover the expected range of isoniazid in the incurred samples [27].

**Figure 3:**
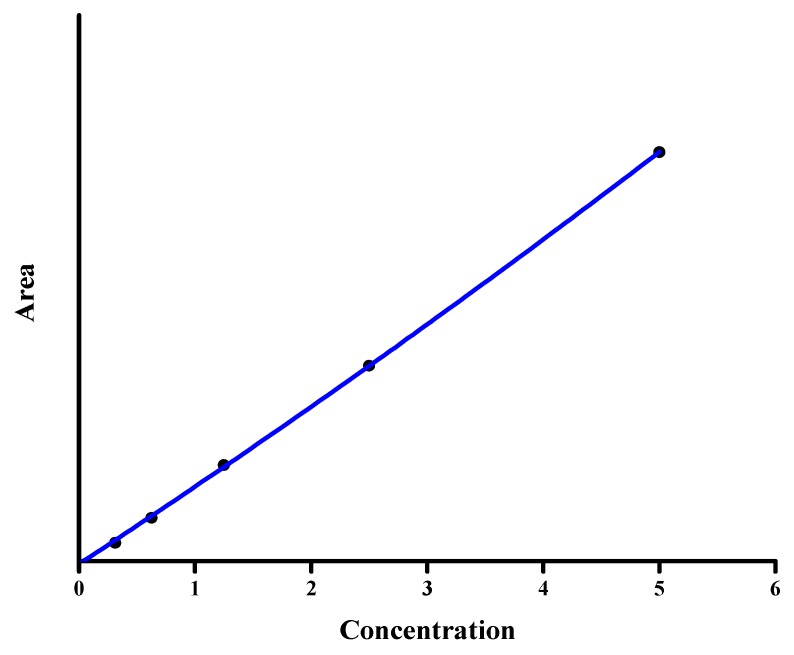
Plot of the peak area vs. the concentration and calibration curve for isoniazid quantification (concentration in μg/mL).

The calibration equation (slope, y-intercept, and determination coefficient) was obtained by plotting the chromatographic peak area of isoniazid versus the concentration using the least-squares linear regression method (Figure 3). To study the variability of the calibration parameters, five curves were obtained by independent measurements on 6 days (one curve each day) over a 3-month period for a different set of standards. A good linear relationship between the independent and observed variables was found. The regression curve, taken as the average of the obtained five calibration curves, was

Peak area = 38,150 ± 21 (isoniazid, μg/mL) + (−4.2 ± 0.3), *r*^2^ = 0.9998.

#### 3.2.3. Detection and Quantification Limit

The limits of detection (LOD) and quantification (LOQ) for isoniazid were determined using the 3 s and 10 s criteria—3.3 and 10 times, respectively. The SD of the lowest concentration solution included in the calibration curve was divided by the slope. The LOD and LOQ of isoniazid were 10 and 30 ng/mL, respectively.

#### 3.2.4. Precision and Accuracy

The intraday and interday accuracy and precision of the method were determined by analyzing isoniazid at three different concentrations: 0.03, 0.25, and 2.5 µg/mL. The intraday analyses were performed by analyzing six spiked urine samples on the same day.

The interday analysis was the average of five measurements of the intraday values taken on 6 days over a 3-month period performed by different analysts and equipment at the same concentrations. The results, expressed as the relative standard deviation (RSD) for precision and the relative error (ε) for accuracy, for both intraday and interday values are shown in Table 1. The values of accuracy (between −0.9 and +8.5%) and precision (RSD less than 16.0%) were below the maximum accepted values according to FDA guidelines (less than 20% for accuracy and precision at the LLOQ, and <15% at higher levels). Thus, the developed procedure can be used in routine analyses of urine samples from tuberculosis patients taking isoniazid medication.

#### 3.2.5. Carry-Over Effect

A urine sample spiked with isoniazid at 5 μg/mL, and, immediately afterwards, a blank urine sample, were analyzed. In this last one, no peak was observed in the chromatogram at the retention time of the isoniazid. Thus, the carry-over was considered negligible at concentrations within the calibration range.

#### 3.2.6. Matrix Effects

The influence of the endogenous compounds of urine in the quantitative results, either by linking or any interaction interfering with the retention process, was evaluated. Working standard solutions containing the same concentration of isoniazid as in Section 3.2.4 divided by five were analyzed by following the same protocol to consider the dilution in the experimental procedure. The results for the intraday accuracy and precision were similar to those obtained for urine samples (Table 1). Therefore, no significant matrix effect was detected, regardless of the complexity of the chemical composition of urine. This was probably done by the interaction of the endogenous compounds of the urine and the SDS-micelles.

#### 3.2.7. Dilution Integrity

The effect of the introduction of another dilution step was investigated. Urine samples spiked at 15 μg/mL were 1/10 diluted and then processed as in Section 2.4. The intraday accuracy and precision were determined (Table 1).

The results were inside the acceptance criteria, and then the method allows a sample over ULOQ to be analyzed after the appropriate dilution. 

#### 3.2.8. Robustness

The robustness of the developed method was observed by slightly changing the chromatographic conditions, like the flow rate, percentage of modifier, concentration of surfactant, etc., one by one, keeping the others at their optimal values (Table 2). A working standard solution of 1 µg/mL was used.

The slight change in the chromatographic parameter did not significantly affect the separation parameter of the selected method; thus, the method can be considered quite robust. As expected, only the variation in the flow rate had an influence on the retention time of the studied compound, whereas the other parameters showed negligible change.

### 3.3. Analysis of Incurred Urine Samples

The efficacy of any treatment depends on the metabolic activity of the drug, i.e., how fast the drug is eliminated from the body. This fact is very important when discussing drugs that have life threatening effects or have a short therapeutic window. Tuberculosis (TB) also falls under the above-mentioned category. If the treatment of the diseases is not properly controlled, it may lead to multidrug resistant tuberculosis (MDR-TB). Since the metabolic rate of the medicine differs for every patient, the plasma concentration of the medicine will also be different in different patients.

Therefore, in the treatment of life-threatening diseases, administrating the correct dose to the patient is very important. Therapeutic drug monitoring (TDM) is an area that helps clinicians or pharmacists to calculate the next dose for effective treatment. So, finally, TDM depends on the analytical method which should be very sensitive, selective, fast, reliable, and inexpensive as well as easy to handle.

**Figure 4:**
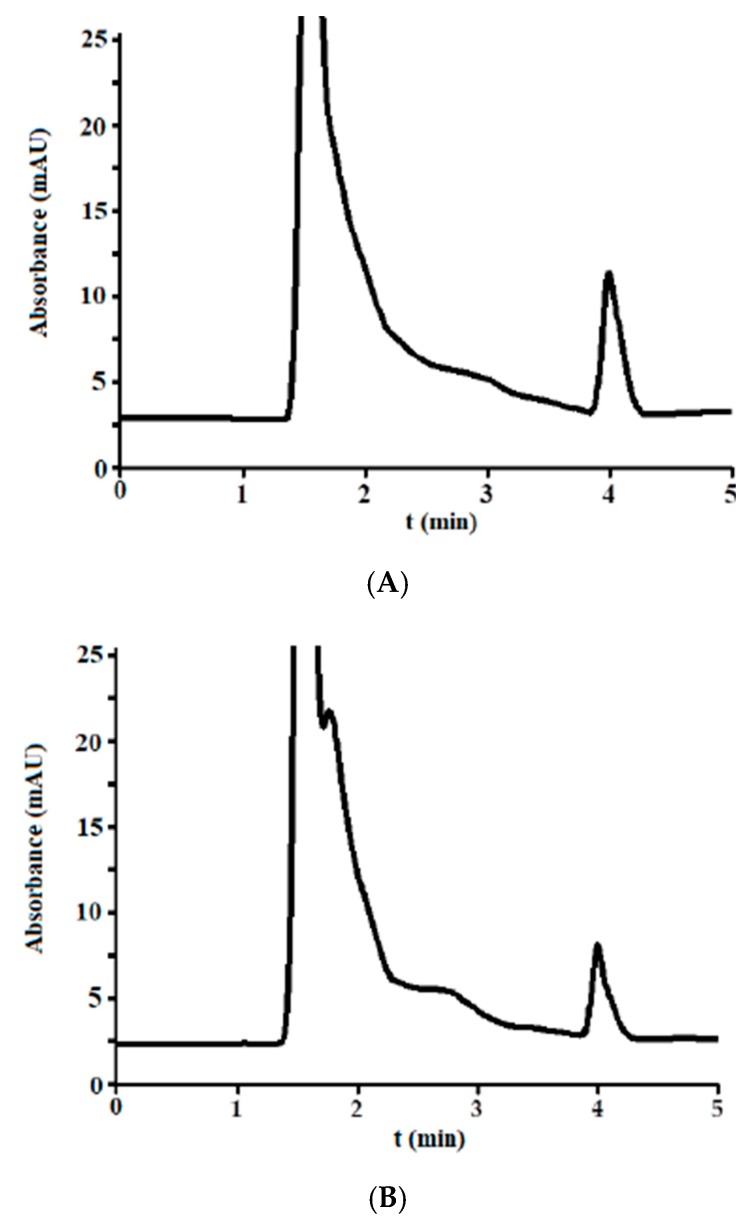
Chromatogram obtained from the analysis of urine from patients 1 (**A**) and 2 (**B**).

The developed method was applied to incurred urine samples from 15 TB patients (Table 3) to detect the presence of isoniazid. The chromatogram showed a similar shape to those obtained in Section 3.1.1, and no overlapping peaks were detected. The chromatogram obtained from analysis of urine from patients 1 and 2 are shown in Figure 4A,B, respectively. The results confirm that the excreted concentration of isoniazid differs from patient to patient.

## 4. Conclusions

The developed method was applied to real urine samples obtained from patients undergoing treatment for TB to determine the level of unmetabolized isoniazid present in urine. Using the developed method, isoniazid was eluted at 4.0 min. The LOD and LOQ were 0.010 µg/mL and 0.03 µg/mL, respectively. The reported method can easily be used for drug monitorization in clinical laboratories in order to improve drug dosages to help with the optimization of tuberculosis treatment. The present method was shown to effectively detect and quantify isoniazid in urine, which is one of the fluids where the unmetabolized isoniazid can be detected and quantified. Detection of isoniazid in urine can act like a marker to determine the isoniazid acetylator status and also to check patient adherence to the treatment of TB. It might also be useful for therapeutic monitoring to determine the required dosages of the drugs in the treatment of TB. The developed method could also be used in the pharmaceutical industry for quality control of isoniazid in different pharmaceutical preparations.

## Figures and Tables

**Table 1:** Intraday and interday accuracy and precision.

Matrix	Concentration (μg/mL)	Intraday ^a^		Interday ^b^	
		Accuracy (ε, %)	Precision (RSD, %)	Accuracy (ε, %)	Precision (RSD, %)
Urine	0.03	+8.5	9.3	+6.9	16.0
	0.25	+3.2	6.6	+2.5	7.5
	2.5	−0.9	2.4	−0.4	2.9
Urine (dilution integrity)	15	+1.9	3.2	-	-
Working solution	0.006	+7.3	7.5	-	-
	0.05	+3.0	5.2	-	-
	0.5	−0.2	2.0	-	-

^a^*n* = 6; ^b^ *n* = 5. RSD: relative standard deviation.

**Table 2:** Robustness evaluation of the developed micellar liquid chromatography (MLC) method (RSD, %).

Chromatographic Condition	Interval	t_R_ (min)	N	B/A
Flow rate (mL/min)	0.9–1.1	12.1	0.9	1.2
pH	6.8–7.2	0.1	2.6	1.6
1-propanol proportion (%)	12–13	0.1	1.5	2.3
SDS concentration (M)	0.14–0.16	0.7	0.7	2.0

**Table 3:** Amount of isoniazid found in the urine of 15 tubercular patients.

Patient Number	Amount of Isoniazid µg/mL
Patient 1	2.9
Patient 2	1.34
Patient 3	4.31
Patient 4	8.45
Patient 5	2.98
Patient 6	3.45
Patient 7	5.34
Patient 8	2.84
Patient 9	7.23
Patient 10	6.89
Patient 11	3.14
Patient 12	5.48
Patient 13	5.39
Patient 14	6.78
Patient 15	3.87
